# The correlation between first words appearance and productive speech in adolescents with schizophrenia

**DOI:** 10.1192/j.eurpsy.2021.1432

**Published:** 2021-08-13

**Authors:** E. Shvedovskiy, N. Zvereva

**Affiliations:** 1 Department Of Clinical Psychology, Mental Health Research Center, Moscow, Russian Federation; 2 Clinical Psychology, Federal State Budgetary Scientific Institution Mental Health Research Center; MSUPE, Moscow, Russian Federation

**Keywords:** speech, schizophrénia, early development, adolescents

## Abstract

**Introduction:**

Shown that there is connection between early development and the current speech parameters in adolescents with schizophrenia. With a more pronounced lag in speech, there was a decrease in the actualization of speech semantic links.

**Objectives:**

Present work aims for a more detailed analysis of the correlations between early speech development and the actual level of development of speech activity in adolescents with schizophrenia.

**Methods:**

Sample

**Table:** 

Age	12,2 - 18,2 (SD=1,35)
males	17
females	13
DS	F20.xx, F21.xx, F25.xx, F06.xx, F32.xx, F33.xx, F50.xx.

Analysis of medical records (medical history) “Syllabic Test”. Parameters: Standard Ratio (SR, SR_2, SR_3); Response Time (RT, RT_2, RT_3). The correlation between the indicators measured by the Spearman correlation coefficient (r_s_).

**Results:**

There was no statistically significant correlation between the First Words (FW) and SR: r_s_ = -0.031, p> 0.05.FW and SR_2 (r_s_ = -0.004, p> 0.05), FW and SR_3 (r_s_ = 0.107, p> 0.05). In addition, statistically significant correlation did not revealed between FW and RT: FW and RT (r_s_ = 0.067, p>0.05), FW and RT_2 (r_s_ = 0.041, p>0.05), FW and RT_3 (r_s_ = 0.066, p>0.05).

**Conclusions:**

The results obtained on the Syllabic test in adolescent sample correspond to the previously identified indicators in adult patients with schizophrenia. RT tends to increase with an increase in the FW age. The limitations of present study: the lack of objectivity in medical history data (mainly parents interview), small sample size and large heterogeneity of DS of patients.

